# The HIV-1 Capsid: From Structural Component to Key Factor for Host Nuclear Invasion

**DOI:** 10.3390/v13020273

**Published:** 2021-02-10

**Authors:** Viviana Scoca, Francesca Di Nunzio

**Affiliations:** 1 Advanced Molecular Virology and Retroviral Dynamics Group, Department of Virology Pasteur Institute, 75015 Paris, France; viviana.scoca@pasteur.fr; 2 BioSPC Doctoral School, Universitè de Paris, 75015 Paris, France

**Keywords:** HIV-1, capsid, nucleus, PIC, RTC, MLO

## Abstract

Since the discovery of HIV-1, the viral capsid has been recognized to have an important role as a structural protein that holds the viral genome, together with viral proteins essential for viral life cycle, such as the reverse transcriptase (RT) and the integrase (IN). The reverse transcription process takes place between the cytoplasm and the nucleus of the host cell, thus the Reverse Transcription Complexes (RTCs)/Pre-integration Complexes (PICs) are hosted in intact or partial cores. Early biochemical assays failed to identify the viral CA associated to the RTC/PIC, possibly due to the stringent detergent conditions used to fractionate the cells or to isolate the viral complexes. More recently, it has been observed that some host partners of capsid, such as Nup153 and CPSF6, can only bind multimeric CA proteins organized in hexamers. Those host factors are mainly located in the nuclear compartment, suggesting the entrance of the viral CA as multimeric structure inside the nucleus. Recent data show CA complexes within the nucleus having a different morphology from the cytoplasmic ones, clearly highlighting the remodeling of the viral cores during nuclear translocation. Thus, the multimeric CA complexes lead the viral genome into the host nuclear compartment, piloting the intranuclear journey of HIV-1 in order to successfully replicate. The aim of this review is to discuss and analyze the main discoveries to date that uncover the viral capsid as a key player in the reverse transcription and PIC maturation until the viral DNA integration into the host genome.

## 1. Introduction

Human immunodeficiency virus 1 (HIV-1) is part of the lentiviruses subfamily that disseminated around humans starting from the twentieth century; nonetheless, the virus was isolated only in 1983 [1]. The main outcome of HIV-1 infection is the deep depletion of CD4^+^ T lymphocytes; however, the count decrease is just transient during the first weeks of infection, making complex the early diagnosis. The T count reduction slowly worsens over the years, yielding to the Acquired Immune Deficiency Syndrome (AIDS) and the related consequences. 

The key feature of lentiviruses consists in the ability to reverse transcribe their RNA genome into double-stranded DNA with subsequent integration into the host chromatin [2,3]. Usually, HIV-1 integration step targets active host genes to ensure the release of its own progeny, but some not yet clear conditions favor the persistence of silent viral genomes (a process known as latency) [4]. Indeed, the virus survives silently in apparently healthy cells, making it difficult to cure AIDS. For their importance in the viral life cycle, the reverse transcriptase (RT) and the integrase (IN) have always been in the spotlight as crucial partners of the reverse-transcribed DNA and as therapeutic targets [5,6]. However, from the past years, the viral capsid progressively gained relevance both in reverse transcription and post-nuclear entry steps [7,8,9,10,11,12], but also as a target for new anti-retroviral treatments [13,14]. Indeed, the scientific community is abandoning the early and absolutistic view of the immediate uncoating, likely arisen from the difficulties in studying the association of viral capsid with the Reverse Transcription Complexes/Pre-integration Complexes (RTC/PIC) by biochemical essays [15,16]. Thanks to new cutting-edge technologies to study the fate of the capsid in infected cells, it has now been put forward the idea of a more tightly regulated uncoating process [11], in which the core shell is preserved until the nuclear translocation step [17]. Importantly, the progressive uncoating ensures protection of the viral complexes from the cytoplasmic environment, and it plays a key direct or indirect role in DNA synthesis, nuclear import, and integration. On one side, in vitro studies are essential for the characterization of the RTC and PIC, being that these transient and heterogeneous viral structures are very difficult to study in cells. On the other side, in vitro studies as biochemical approaches of purification of RTC/PIC [15,16,18] were unable to unravel the role of the capsid in HIV-1 life cycle. These results were mainly due to the cell-free experimental context or for the usage of strong detergents. In recent years, the advent of new technologies, particularly in the imaging field, allowed to develop a better overview of the role of the viral capsid, not only for its structural importance, but also as a key viral component for the RTC/PIC dynamics in the cells. In this review, we aim to describe the new studies which are allowing to uncover the importance of HIV-1 capsid in viral early life cycle steps, besides its structural role.

## 2. Analysis of HIV-1 Core Dynamics along the Reverse Transcription Process in Cell-Free Conditions

Both the discovery of the human oncogenic retrovirus, HTLV-1 [19] and of HIV-1 [1] were based on the detection of RT activity in cells derived from infected patients. From that discovery, several groups aimed to unravel the composition of the RTC and its functional dynamics in vitro and in cells [20]. First hints about the impact of the core proteins in the reverse transcription process were found through the employment of CA N-term domain mutants implied in low- or high-core stability [21,22,23]. Indeed, the premature uncoating or any impairment of the CA multimeric lattice highly affects viral early life cycle steps, in particular the initiation of the DNA synthesis. These observations mainly derive from studies showing the interplay between the viral CA and host co-factors, such as IP_6_ [24,25] and Cyclophilin A (CypA) [26,27,28] or restriction factors, like TRIM5α [29], and synthetic molecules, such as PF74 [30,31], which all specifically target HIV-1 capsid and affect the outcome of reverse transcription. It has been observed that a premature uncoating inhibits the reverse transcription process [29], thus the stability of the viral core has a direct impact on reverse transcription efficiency [23], disproving the model of immediate uncoating. Nonetheless, the cores evidently remodel during the journey to the nucleus, not only due to the cellular environment, but also due to the reverse transcription process. Cosnefroy et al. proposed that this type of remodeling starts specifically after the first strand transfer [32]. Experiments of in vitro reverse transcription paired to time-lapse atomic force microscopy show how the newly synthetized DNA increases the pressure inside the core, which triggers the disassembly [33]. In summary, the capsid is a permeable and dynamic structure that persists along the whole reverse transcription process as intact or partial core. In support of this hypothesis, very advanced results in a cell-free system show that a partial rupture of the core is caused by the dsDNA synthesis, but the majority of the DNA genome is packed inside [34].

## 3. HIV-1 Capsid as Crucial Nuclear Import Partner of the RTC/PIC

During the early steps of the viral life cycle, the HIV genome begins to be reverse transcribed to generate both episomal forms and a mature PIC, the latter contains the essential components for viral integration: the fully reverse-transcribed DNA accompanied by the viral IN. In the last few years, biochemical and cellular studies have contributed to reveal the detailed RTC/PIC structure with the identification of viral [18,35,36,37,38] and host factors, such as lens-epithelium-derived growth factor (LEDGF/p75), barrier-to-autointegration factor (BAF), high-mobility group proteins (HMGs) [39,40,41,42,43,44]. The role of capsid in PIC dynamics became clear with its relevance in HIV-1 DNA nuclear import, especially along the search of determinants of non-dividing cells infection. Pioneer studies had highlighted only the matrix (MA) and Vpr as viral partners of the PIC for the nuclear import [15,45,46,47] and, later on, the central cis-acting DNA flap [48]. The replacement of HIV-1 capsid with the one from Murine Leukemia Virus (MLV) [49] clearly revealed a functional and unique role of HIV-1 capsid in PIC nuclear entry. This replacement impairs nuclear import in non-mitotic cells [49], indeed the infection of non-mitotic cells is a peculiarity of lentiviruses, thus HIV-1 capsid evolved to successfully infect this type of cells [7,9].

HIV-1 capsid is indeed the main intermediator between the RTC/PIC complex and the cellular factors involved in nuclear import, like the nucleoporins Nup358/RanBP2 [50,51] and Nup153 [52,53,54], which are critical host factors for viral nuclear invasion. More specifically, Nup358/RanBP2 directly interacts with the Cyp-like domain of the viral CA allowing the docking of the RTC/PIC to the cytoplasmic side of the Nuclear Pore Complex (NPC) [50]. Then, Nup153, which is exclusively part of the nuclear basket of the pore, binds to multimeric CA in its hydrophobic pocket and aids the RTC/PIC to translocate through the NPC channel [52,53,54,55,56]. Interestingly, Nup153 is also involved in the nuclear import of the genome of other HIV-unrelated viruses. The yeast nucleoporin, Nup124p, ortholog of the human Nup153, binds the Tf1 Gag protein enhancing the nuclear import of the retrotransposon Tf1 [57]. Nup153 also interacts with the HBV capsid [58], indicating that Nup153 may be a common partner among some viruses that exploit the nuclear compartment.

Other host factors like TNPO3 have been found to be indirectly involved in HIV-1 nuclear import [59,60,61,62,63] affecting the localization of CPSF6 (Cleavage and Polyadenylation Specific Factor 6) [64,65,66], a polyadenylation factor, whose role in HIV-1 early steps is still under investigation. CPSF6 directly binds the viral CA and it is suggested that this interaction facilitates the translocation of HIV-1 PIC in the nucleus [55,67,68,69]. Additionally, it has been demonstrated that the depletion of CPSF6 or the infection with HIV-1 CA mutants, defective for CPSF6 binding, alter viral integration site selection [69,70,71]. However, in some kinds of cells depleted for CPSF6, the viral infectivity was not reduced, thus the relevance and the mechanism behind the CPSF6 role require further studies. The relevance of CA/CPSF6 binding has been investigated in detail by Yamashita laboratory [72]. They exploited HIV-1 CA carrying the point mutation A77V that showed a reduction in CPSF6 binding, while this single amino acid mutation does not affect the late steps of viral life cycle. Using animal models, they found that HIV-1 CA mutants reverted to the wild type CA. This result highlights that, although CA/CPSF6 binding is not essential for HIV-1 replication, this host–viral interaction confers a significant advantage to the viral fitness [72]. On the other hand, ex vivo experiments published by Kräusslich group showed that the depletion of CPSF6 in macrophages led to accumulation of viral complexes at the nuclear envelope followed by a reduced infectivity [56]. Controversially, the group of Melikyan has described in a recent manuscript that the CA/CPSF6 interaction is largely dispensable for HIV-1 infection in macrophages, yet the lack of this host–viral interaction excludes viral genomes from nuclear speckles (NSs), leading the viral integration in non-canonical sites [73]. All the aforementioned studies implied the investigation and the visualization of the viral IN or CA, often using surrogated viruses, or by labeling the reverse-transcribed genome with approaches not compatible with single-molecule visualization.

New technologies will provide more detailed and less artificial information about the viral life cycle in both dividing and non-dividing cells, with the aim of imaging RTC/PIC complexes during HIV-1 infection. To this purpose, a bipartite system derived from a bacterial ParABS chromosome segregation machinery has been recently adapted to visualize HIV-1 DNA. This system is known as HIV-1 ANCHOR [17]. The HIV-1 genome has been modified to carry a limited number of nucleation parS sites (ANCH sequence) to which modified ParB proteins (OR) bind and then spread onto adjacent DNA through a mechanism of protein–protein interaction [74,75,76], which amplifies the signal allowing single-DNA detection [77]. Through the coupling of HIV-1 ANCHOR technology [17,77] to track the HIV-1 DNA, and the electron microscopy for the detection of the CA (Correlative Light-Electron Microscopy (CLEM)), Blanco et al. pinpointed the PIC crossing the nuclear pore containing multiple CA proteins [17] in a quasi-wild type HIV-1 infection context. In particular, immunogold-labelled CA structures were revealed thanks to the exposure of epitopes on the sides of the analyzed sections. Interestingly, a different distribution of gold particles between the cytoplasm and the nucleus has been observed. This difference reveals the presence of divergent CA shapes in the cytoplasm compared to the nucleus, indicating a core remodeling during viral nuclear entry [17].

This observation merges the two concepts of the core remodeling, necessary to cross the NPC channel, and the capsid relevance in nuclear transport, in single-cell.

## 4. HIV-1 CA Involvement in Post-Nuclear Entry Steps

The ability of HIV-1 to cross the NPC is addressed to the interplays between CA proteins and host factors at the cytoplasmic/nuclear interface, both in mitotic and non-mitotic cells. However, what happens to the capsid right after nuclear entry? Is the role of capsid limited to PIC transport? Is the viral capsid associated to the viral genome inside the nucleus? HIV-1 nuclear import is tightly bridged to integration [52,78,79]; thus, the capsid could be directly or indirectly involved in the steps that follow the NPC passage.

### 4.1. The Role of the HIV-1 CA in the Nucleus

The presence of HIV-1 CA in the nucleus was firstly suggested by the in vitro interaction of assembled cores with Nup153, exclusively located in the nuclear side of the NPC [52,53,78] and by experiments on the impairment of nuclear import conditions [62,80]. Next, thanks to the employment of surrogate viruses, it was possible to detect and live-track nuclear CA proteins, in different cell types [73,81,82,83]. However, to decipher whether the CA proteins can drive the viral genome through the NPC, it has been essential the coupling of the CA detection to a reliable DNA labelling technology, to study CA-PIC structures during and after nuclear import. This has been recently demonstrated by Blanco et al., who exhibited ultrastructural imaging data, showing CA complexes in the nucleus associated to the viral DNA [17]. These results were obtained by the coupling of the immunogold labeling to fluorescence microscopy by CLEM [17]. In addition, experiments of double-gold labeling indicated the intra-nuclear presence of complexes formed by CA proteins associated to the viral DNA [17]. On the other hand, confocal microscopy also revealed HIV-1 CA association with the viral DNA detected by EdU labelling, which is based on a metabolic labeling of DNA with “clickable” nucleoside analogs [84]. EdU labeling represents an important strategy to track down newly synthetized DNA, in non-dividing cells or mitotically blocked cells [85]. Using this DNA labeling technique, Peng et al. were able to study by imaging RTCs/PICs carrying HIV-1 DNA labelled with EdU. A major difference was highlighted between HeLa and monocyte-derived-macrophages (MDMs) in the detection of nuclear CA, which was much stronger in macrophages [86]. Indeed, studies using imaging technologies with a resolution higher than that imposed by the diffraction limit of light, such as super-resolution structured illumination microscopy (SIM) or photoactivable localization microscopy (PALM) [87], had confirmed the low CA amount in HeLa cells nuclei [82]. Proliferating cells, like HeLa and activated CD4+T lymphocytes, may be more prone to lose nuclear capsid multimers than macrophage cells, because of cellular division or because of a faster HIV-1 replication. Moreover, it is likely that this cell type-dependency is given by differences in cellular determinants that are involved in HIV-1 core stability [88], as well as by the reverse-transcription dynamics in dividing and non-dividing cells [85,89]. A recent study from Kräusslich group suggested that CPSF6 shields CA epitopes, explaining the difficulties of CA detection in the nucleus of CD4+ T cells by immunofluorescence [90]. However, Chin et al. were able to fluorescently label the viral CA in dividing and non-dividing cells, like HeLa, U2OS and MDM cells, in co-localization with the HIV-1 DNA labeled with ViewHIV technology in fixed-cells [67]. Burdick et al. showed that a large viral CA signal derived from viruses incorporating enhanced green fluorescent protein (eGFP)-tagged capsid (CA-eGFP) proteins [91] can be detected in the nucleus only before viral transcription, suggesting that an intranuclear uncoating occurs near HIV-1 integration sites [81]. Newly published data show that multiple viruses accumulate, conveyed by CPSF6, in speckle organelles in macrophages [73,85], which contain a major amount of viral nuclear CA in comparison to T cells, facilitating the nuclear CA visualization by confocal microscopy. More research is required to assess if dividing cells concentrate less CA-positive viral complexes in the nucleus, compared to terminally differentiated ones. The heterogeneity of the data on capsid nuclear detection has been also addressed to the differences in antibodies used [67]. This issue can be unraveled by electron microscopy studies; indeed, using this technology, it has been shown viral CA complexes in the nucleus regardless of the employed antibody, in HeLa cells and CD4+ T lymphocytes [17]. Cryo-EM studies have also been performed in infected macrophages to reveal viral CA structures in the nucleus [90].

New insights into the functionality of HIV-1 CA in the nucleus were only proposed during 2020, and it is slowly becoming possible to solve the puzzle of HIV-1 early steps triptych: reverse transcription, uncoating, and integration. An interesting biochemical study confirms the presence of HIV-1 CA oligomers in the nucleus using hyperstable cores allowing reverse transcription, showing that the majority of reverse-transcribed intermediate products are enriched in the nuclear fractions [27]. Along the same lines, recent studies highlighted that the process of reverse transcription can occur in the host nucleus. Newly synthetized viral DNAs cluster in the nucleus, colocalizing with incoming viral RNA [85]. Indeed, the viral RNA accumulates in the nucleus upon block of RT through a reversible inhibitor, the Nevirapine. When the drug is washed out, the reverse transcription is restored, generating newly synthesized viral DNA, highlighting the occurrence of nuclear reverse transcription [85]. Another recent study, exploiting NPC blockade, showed that HIV-1 crosses the nuclear envelope (NE) in less than 5 h post infection [92] and supports that completion of both reverse transcription and uncoating follow the nuclear import. Taken together, all these results imply that partial or intact cores containing the viral genome can reach the nuclear compartment and synthetize DNA inside the nucleus.

### 4.2. The Role of the HIV-1 CA in the Viral Integration

**Figure 1:**
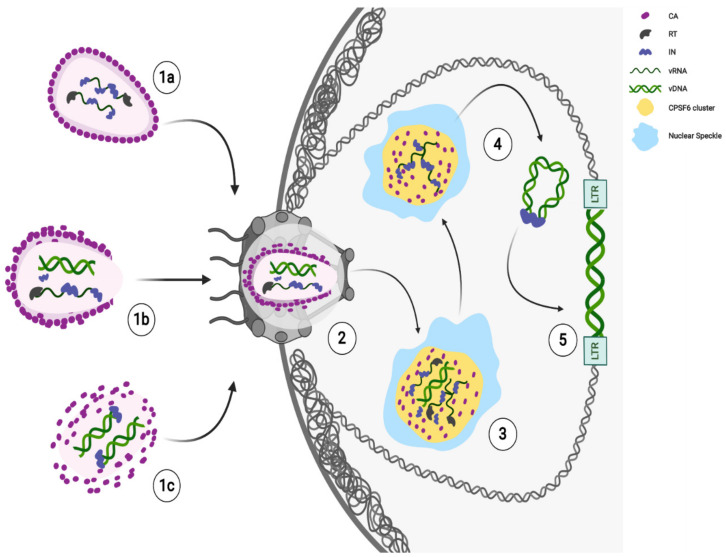
Ongoing model of HIV-1 early steps. HIV-1 cores are released in the cytoplasm after membrane fusion. Likely, the core state reflects different stages of reverse transcription. Different potential core states can reach the nucleus: nearly intact (**1a**), remodeled (**1b**), or partially uncoated (**1c**). Next, HIV-1 core remodels to translocate through the nuclear pore complex (NPC) channel and CA proteins interact with Nucleoporins (Nup358/RanBP2, Nup153) for the translocation of RTCs/PICs (**2**). The completion of reverse transcription and maturation of functional PICs (**3**) occur in HIV-1 MLOs, thanks to the CPSF6 clusters formation in SC35 nuclear speckles (NSs). HIV-1 mature PICs separate from HIV-1 MLOs (**4**); as long as incoming viral RNA is retained inside, viral components, like the IN proteins remain accumulated in the clusters (**4**). Integration may occur in active chromatin regions in proximity of the nuclear speckles and of the nuclear envelope (**5**). Created with BioRender.com.

A role of the CA in HIV-1 integration has been envisaged as the depletion of the Nups capable of binding the viral CA hexamers results in a change in the distribution of integration sites [52]. However, it is still not clear whether the viral CA plays a direct role in the HIV-1 integration. Probably, nuclear uncoating could represent an advantage for HIV-1 nuclear steps. Viral CA complexes can protect the viral genome from antiviral sensors and escort the mature PIC to the vicinity of active gene regions for efficient viral integration. Following in live HIV-1 labelled CA in HeLa cells, it is possible to see the disappearance of CA signal at the nuclear location where an HIV-1 transcriptional focus appears [81]. Then, how does HIV-1 CA know where to locate the viral genetic material? CPSF6-CA interaction [68] seems to dictate the nuclear location of HIV-1 genomes [73]. Importantly, through the direct detection of HIV-1 DNA, it was possible to visually confirm the relevance of this interaction, which might have a role in HIV integration sites distribution [69,70,71,93]. New results demonstrate that EdU-labelled viral DNA [85], as well as viral IN [73,77], accumulate in CPSF6 clusters, which are retained in SC35-positive nuclear speckles [73,77]. Therefore, CPSF6 seems to be not only responsible for HIV-1 CA shuttling, but also to escort the RTC/PIC in nuclear speckles (NS) regions where the viral DNA labeled with EdU has been found [73,85]. Of note, the NS regions are known to be interchromatin granules [85,94]; therefore, they unlikely can be sites of viral integration. However, NSs have been indicated as HIV-1 integration sites because of the detection in these nuclear organelles components of the P-TEFb complex [73], which are usually recruited to the transcribing viral genome [95]. On the other hand, these factors were found by other authors in the vicinity of NSs [94]. NSs are also considered nuclear storage sites, rather than sites of functional processes [94,96]. However, what determines the specific subset of transcription factors localized to nuclear speckles is still unclear. Mainly, NSs are important for the assembly of higher-order complexes and/or for the state or accessibility of splicing and transcription factors [94]. Indeed, NS-neighboring chromatin regions that contain active chromatin [97] represent favorable chromatin loci for viral integration [77] (Figure 1).

Recent findings show late HIV-1 reverse-transcribed DNA separated from IN foci [77,90] in the nucleus. The majority of intranuclear-detected INs are retained in CPSF6 clusters, while the integration of the viral DNA occurs in proximity, but not inside these nuclear condensates [77] (Figure 1). Thus, the HIV-induced CPSF6/SC35 membraneless organelles (HIV-1 MLOs) might be safe nuclear sites to complete reverse transcription and uncoating. Again, the accumulation of RTC in CPSF6/NS appears to precede the completion or, possibly, the beginning of DNA synthesis. Concentration of forming PICs in NS may promote integration of HIV-1 into euchromatin regions located outside, but not far from NS, for optimal replication [77].

## 5. Conclusions

The knowledge of HIV-1 capsid function deeply evolved from the mere structural-shield protein. Recent exciting results about nuclear uncoating and nuclear reverse transcription completely change our view on HIV-1 early phases of the life cycle. Apparently, nuclear import precedes the complete uncoating and the reverse transcription can occur in nuclear HIV-specific membraneless organelles (HIV-1 MLOs), at least in macrophages. Does the nuclear reverse transcription represent an advantage for HIV? Is it correlated to an efficient replication? All these questions open new frontiers of research based on the role of HIV-1 MLOs on viral persistence and rebound, which represent the major obstacle to cure AIDS.

Possibly, the nuclear CA can trigger nuclear antiviral pathways [98], but a late uncoating keeps safe the viral genetic material until integration. Future studies are required to clarify whether the viral CA may play a direct role in choosing integration sites or whether viral CA is acting behind the scenes to direct virus integration. Surely, new single-cell cutting-edge technologies are allowing and will continue to allow to build a new model of HIV-1 early steps (Figure 1).
